# Terra Firma-Forme Dermatosis: A Heart-Shaped Dark Lesion in a Boy

**DOI:** 10.7759/cureus.59565

**Published:** 2024-05-03

**Authors:** Axel Rodrigo Márquez Núñez, Leticia Boeta Ángeles

**Affiliations:** 1 Dermatology, National Autonomous University of Mexico, Mexico City, MEX; 2 Dermatology, Hospital "Juárez Centro", Mexico City, MEX

**Keywords:** hyperpigmentation, dermatosis neglecta, acanthosis nigricans, dermatoscopy, terra firma-forme dermatosis, duncan dirt

## Abstract

Terra firma-forme dermatosis is an acquired and idiopathic disorder with an underestimated incidence. It is characterized by brownish skin pigmentation, forming asymptomatic plaques that give a soiled skin appearance. Soap and water have a minor effect; however, friction with 70% ethyl or isopropyl alcohol immediately eliminates plaques to a normal skin appearance, thus being the ideal method for diagnosis and treatment. The lack of familiarity with this disease possibly contributes to an alarming underdiagnosis. In this report, the authors present a case of terra firma-forme occurring in a 14-year-old Mexican patient who presented with a heart-shaped pigmented lesion in the pubic area.

## Introduction

Terra firma-forme dermatosis, named by Duncan et al. in 1987 [1], has been regarded since its origins as a cosmetic problem that potentially leads to unnecessary diagnostic and therapeutic procedures. This dermatosis is a benign condition, often asymptomatic, that manifests either locally or disseminated. Its hallmark lesions are rough brown-to-black hyperkeratotic plaques of multiple sizes, which might sometimes be referred to as “dirtlike lesions” despite proper hygiene and manual prodding [1-3]. The etiology of this entity remains unknown, although it has been hypothesized to be due to a late maturation of keratinocytes and retention along melanin within the epidermis [3]. Dermoscopy shows numerous polygonal brown clods arranged in mosaic pattern [4]. Differential diagnosis includes acanthosis nigricans, epidermal nevi, tinea versicolor, tinea nigra, reticulated papillomatosis, and post-inflammatory hyperpigmentation [5].

## Case presentation

**Figure 1:**
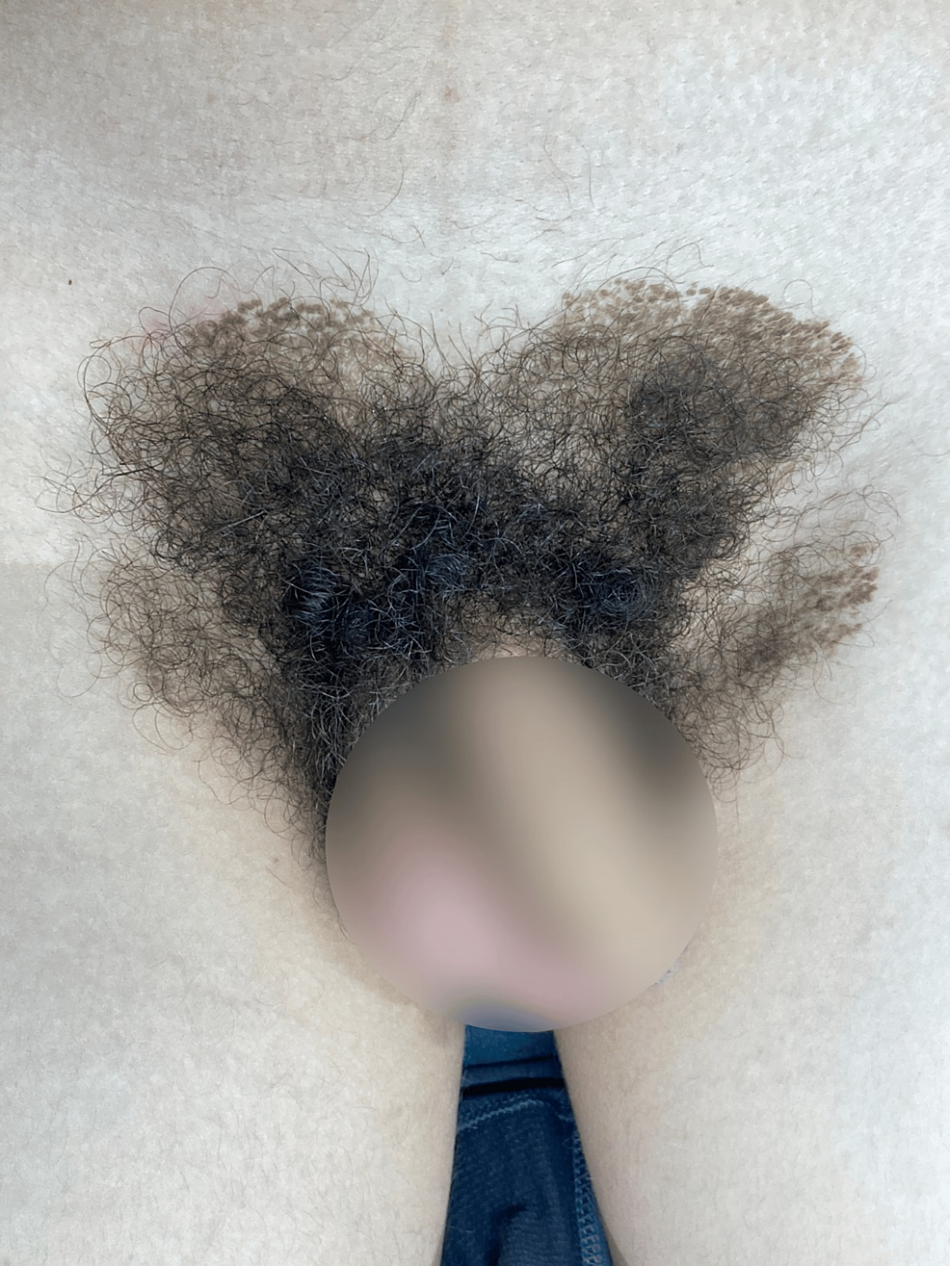
Dermatosis located at the pubic area. Hyperpigmented and hyperkeratotic plaque with a heart-shaped distribution.

**Figure 2:**
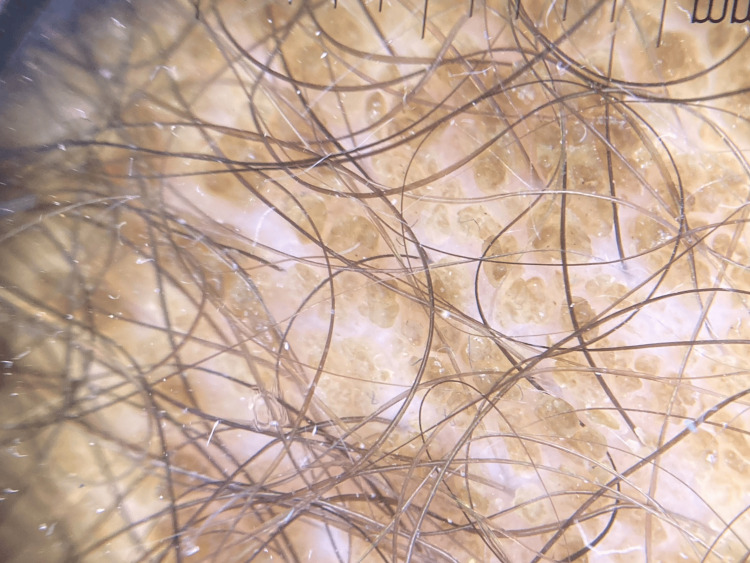
Dermoscopy. Numerous polygonal brown clods arranged in a mosaic pattern.

**Figure 3:**
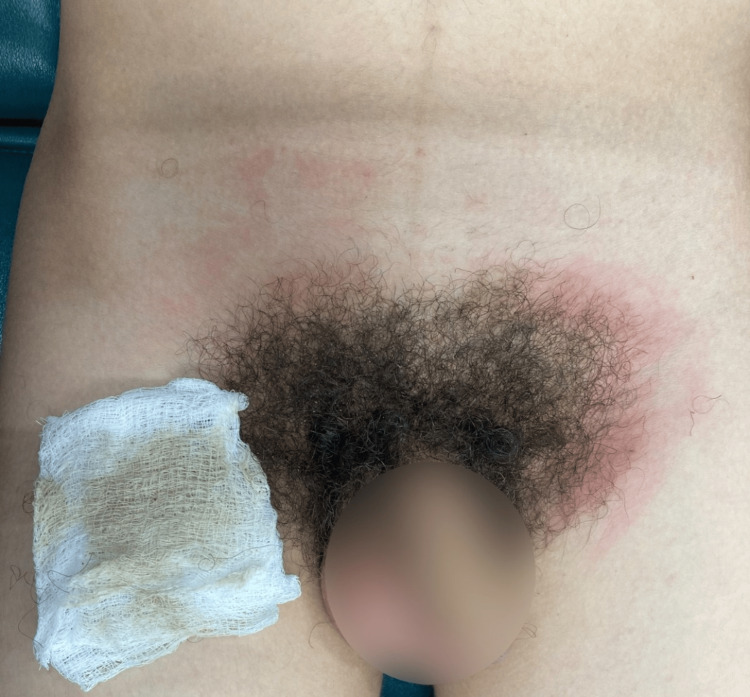
Image after rubbing the dermatosis area with a gauze soaked in 70% ethyl alcohol. Lesions were eliminated and revealed normal-appearing skin.

A 14-year-old male presented to the clinic seeking advice for a skin finding. His past medical history was relevant for blunt genital trauma without evidence of significant changes on ultrasound. Physical examination revealed a 14x12 cm brown hyperpigmented and hyperkeratotic plaque with a heart-shaped distribution in the pubic area. The surface was rough with clear borders, and the patient did not mention associated symptoms. The patient first noticed such plaque 14 days before the initial consult and mentioned skin appearance to persist despite soap scrubbing (Figure 1). Dermoscopy evidenced numerous polygonal brown clods arranged in mosaic pattern (Figure 2). A terra firma-forme dermatosis was suspected, and a friction maneuver was performed with a sterile gauze soaked in 70% ethyl alcohol. This test eliminated the lesions and revealed normal-appearing skin (Figure 3).

## Discussion

Terra firma-forme dermatosis is a benign condition of an unknown origin. The name appears to be originated from the Latin word “terra firma,” which means “dry soil,” and implying as such the hyperkeratosis and hyperpigmentation with a characteristic soiled skin appearance [1]. The clinical presentation can be broad and poses several differential diagnoses including acanthosis nigricans, pseudoacanthosis nigricans, psoriasis, factitious dermatitis, epidermic nevi, tinea versicolor, tinea nigra, reticulate papillomatosis, and post-inflammatory hyperpigmentation [5]. Skin changes appear as brown-to-black plaques of multiple sizes and can affect several body areas. Aslan et al. conducted a retrospective analysis of 79 patients, where a high prevalence (88.6%) in children was found, with a mean age of 10 years at presentation [6]. Initially, the condition affects the neck and trunk skin, and around 25% of patients had an affected limb, with less than 3% affecting head skin. The affected site and disease duration in regard to the morphological condition is not well defined. Skin folds might promote a more rapid sebum, dirt, and debris buildup, favoring a hyperkeratotic presentation [7]. Similarly, disease duration might be directly correlated with a papillomatous morphology, especially if favored by local occlusive factors [7].

Diagnosis is based on recognition of typical clinical characteristics. To confirm this, the gold standard is an attempt to remove skin changes using ethanol or isopropanol alcohol [1-7]. As in other skin diseases with soiled appearance, dermoscopy can provide a diagnostic aid; patients usually show numerous polygonal brown clods arranged in a mosaic pattern [4]. In an analysis of different dermoscopic patterns conducted by Elmas et al., the mean reported age was 19 years, with a majority of males (n=6). They observed that the most common dermoscopic finding was numerous polygonal brown clods arranged in a mosaic pattern (n=7) [4]. Owing to the mostly favorable prognosis of this entity and pediatric population incidence, the need for tissue biopsy is rare [5-7]. Common pathological findings consist of lamellar hyperkeratosis, orthokeratosis, and keratin goblets in the stratum corneum [7-9].

Terra firme-forte dermatosis is instantly treated by rubbing the skin with an 70% ethanol or isopropanol-soaked gauze [1-7]. This clearing, not observed with soap and water, can be interpreted as the main diagnostic sign. However, skin irritation is a risk to be considered when rubbing with ethanol; therefore, a chemical peeling with an alcoholic base should be considered for an improved cosmesis [3-7]. Constant moisture and general skin care on affected sites are the cornerstone to prevent xerosis after isopropanol or ethanol interventions [7-10].

## Conclusions

Terra firme-forte dermatosis is an underestimated common adult and pediatric skin disorder. Diagnosis is clinical, based on an ethanol friction maneuver. This dermatosis should be considered in all patients presenting with soiled skin pigmented lesions, regardless of the location. We present the case of a 14-year-old male with a pubis heart-shaped hyperpigmented skin change that was cleared immediately and completely by rubbing ethanol in the skin area, therefore contributing to the body of knowledge of this entity.
